# Gender differences in acute myocardial infarction—A nationwide German real‐life analysis from 2014 to 2017

**DOI:** 10.1002/clc.23662

**Published:** 2021-06-01

**Authors:** Leonie Kuehnemund, Jeanette Koeppe, Jannik Feld, Achim Wiederhold, Julia Illner, Lena Makowski, Joachim Gerß, Holger Reinecke, Eva Freisinger

**Affiliations:** ^1^ University Hospital Muenster, Cardiol., Dept. of Cardiology I ‐ Coronary and Peripheral Vascular Disease, Heart Failure Muenster Germany; ^2^ Institute of Biostatistics and Clinical Research University of Muenster Muenster Germany

**Keywords:** acute coronary syndrome, epidemiology, gender, health care research, multivariable logistic regression analysis, women

## Abstract

**Background:**

Female sex was reported to be associated with an unfavorable outcome in acute myocardial infarction (AMI). In this nationwide analysis we assessed sex differences in acute outcomes of AMI and recent trends in patient healthcare.

**Methods:**

We analyzed 875 735 German cases hospitalized with a main diagnosis of ST‐ (STEMI) and non ST‐elevation myocardial infarction (NSTEMI) between January 01 2014 and December 31 2017 regarding morbidity, in‐hospital mortality and treatments. A multivariable logistic regression model was designed to evaluate the use of interventions and their impact on in‐hospital mortality.

**Results:**

STEMI cases decreased from 72 894 in 2014 to 68 213 in 2017, with 70% assignable to men. Female sex was associated with older age (74 vs. 62 years), and higher prevalence of cardiovascular risk factors such as chronic kidney disease (19.2% vs. 12.5%), hypertension (69.0% vs. 65.0%) and left ventricular heart failure (36.0% vs. 32.1%). In NSTEMI, female sex was also associated with older age (78 vs. 71 years), and higher prevalence of cardiovascular risk factors such as chronic kidney disease (29.7% vs. 23.9%), hypertension (77.4% vs. 74.5%) and left ventricular heart failure (40.5% vs. 36.4%). Overall, 74.3% of female and 81.3% of male STEMI cases received percutaneous coronary intervention (PCI, p < 0.001). In NSTEMI, PCI was performed in 40.8% of female and 52.0% of male cases (p < 0.001). In‐hospital mortality was notably increased in female patients with STEMI (15.0% vs. 9.6%; p < 0.001; OR 1.07; 95% CI 1.03–1.10) and NSTEMI (8.3% vs. 6.3%; p < 0.001; OR 0.91; 95% CI 0.89–0.93) compared to males.

**Conclusions:**

Our nationwide real‐world data document that in‐patient STEMI cases continue to decrease in women and men. The observed higher in‐hospital mortality in women was largely attributed to a more unfavorable risk and age distribution rather than to female‐intrinsic factors. Women with AMI continue to be less likely to receive revascularization therapies.

## 1 INTRODUCTION

Acute myocardial infarction (AMI) is still among the most common causes of death in men and women in industrialized nations.^1^ Differences related to patients' sex have been reported for incidence, symptom presentation, pathophysiological characteristics as well as treatment strategies and outcome.^2^, ^3^ However, awareness of the risk for heart disease in women is much lower than in men.^4^ Some studies have shown a negative association between female gender and outcome after AMI: specifically women experience more often delays to reperfusion, adverse events such as major bleeding events and complications associated with vascular access.^5^, ^6^, ^7^

Many of the mentioned differences regarding therapy and complications have been attributed to the older average age of female patients at onset of AMI.^2^, ^3^, ^7^, ^8^, ^9^, ^10^, ^11^ So far, women are mostly underrepresented in guideline‐changing cardiovascular disease research, often presenting less than a third of enrolled patients.^12^

In numerous studies, the interaction between gender, risk factors and incidence of myocardial infarction remains controversial.^13^ Some studies have suggested specific gender‐intrinsic‐causes even after adjustment for age and other risk factors,^2^, ^8^, ^14^ while others have failed to establish an association between gender and mortality.^15^, ^16^, ^17^

As the primary objective of this routine‐data‐based analysis, sex differences of recent nationwide trends in in‐patient healthcare and in‐hospital outcome of AMI were evaluated. Furthermore, differences between both sexes of the association of comorbidities and in‐hospital death were investigated to shed additional light on whether the increased mortality in women is due to a more unfavorable comorbidity of women or a potential gender‐intrinsic factor.

## 2 METHODS

The German remuneration system requires the coding of a main diagnosis for all in‐hospital cases. This principal diagnosis must obligatorily reflect the underlying reason for hospitalization. In addition, an unlimited number of additional diagnoses can be coded in order to document coexisting morbidities as well as complications. The diagnoses must be coded in accordance to the *German Modification of the International Statistical Classification of Diseases and Related Health Problems 10th Revision (ICD‐10‐GM)*. Diagnostic, endovascular, and surgical procedures are coded in detail according to the German Procedure Classification (OPS). Diagnoses and procedures assigned to the respective ICD/OPS codes used in this analysis are listed in supplemental Table S1.

Due to federal law, all German hospitals are required to transfer the collected data on all in‐hospitalizations to the national institute for hospital remuneration (*Institut fuer das Entgeltsystem im Krankenhaus*, InEK; Siegburg, Germany; http://www.g-drg.de) since 2002. Thanks to the Federal Statistical office, these large data records are available for scientific purposes after 2 years.

The data analyzed by the study at hand were given by administrative database base provided by the Federal Statistical Offices (Research Data Centre of the Federal Statistical Office and the Statistical Offices of the Länder (Statistisches Bundesamt [DESTATIS]; https://www.destatis.de)) contains all in‐patient treated patients on a case base per year, except for treatments in psychiatric or psychosomatic units. There was only a remote access to anonymous data.

We identified all cases with a main diagnosis of ST elevation myocardial infarction (STEMI) and non‐ST elevation myocardial infarction (NSTEMI) between Jan 1st 2014 and Dec 31st 2017 (using an ICD code I21* or I22* within 28 days after onset of symptoms). Further, data on concomitant diseases, risk constellations and selected cardiovascular procedures were acquired for sex‐specific analysis. Covariables were determined by ICD codes as well, which were only available from inpatient diagnoses codes from the myocardial infarction hospitalization. Further details have been described previously.^18^

### 2.1 Statistics

The analysis covers all in‐patient AMI cases in Germany and does not represent a subsample. All analyses were done for STEMI and NSTEMI cases separately. With regard to the primary question of this work, we want do address differences between female and male patients on in‐hospital mortality, and differences between both sexes in the association of different factors for death in in‐patient STEMI and NSTEMI cases. Furthermore, we want to address different in‐hospital treatment strategies between female and male sex. Relative frequencies of death, in‐hospital stroke, coronary intervention or surgery were tested via two‐sided exact Fischer's test or via two‐sided Chi‐square test, in cases of large amount of data. Multivariable logistic regression analysis for in‐hospital mortality was performed to evaluate the association of sex and in‐hospital death adjusted by patient's risk profile in a full model (including all patients) and for female and male sex separately. The models included age, diabetes mellitus (DM), chronic heart failure (CHF), chronic kidney disease (CKD), peripheral arterial disease (PAD), atrial fibrillation and/or flutter (Afib), hypertension, previous stroke, dyslipidemia, obesity, smoking, cancer and a disjoint categorical variable (no angiography, diagnostic angiography only without revascularization, PCI, CABG) to take account for different treatment strategies.

In order to address different patients risk profiles between female and male sex, we evaluated the interaction of gender with all other variables in the respective full models. P‐values for the test of interaction of all models were jointly adjusted using Benjamini‐Hochberg procedure^19^ to control the false discovery rate (FDR) with respect to the multiple comparison problem. FDR‐corrected p‐values p^int^ ≤ .05 will be compared with the overall significance level of 5%. Unadjusted and FDR‐corrected interaction p‐values for all models are presented in the supplements (supplemental Table II). All other p‐values, i.e. the main effects in the full models, the effects in the subgroups or presented frequencies of in‐patient outcome are purely descriptive and unadjusted. Odds ratios (OR), unadjusted 95% confidence interval (CI) and unadjusted p‐values for all variables in the subgroups are shown in (Figure 4; supplemental Table IV). Inferential statistics are intended to be exploratory (hypotheses generating), not confirmatory, and are interpreted accordingly. Statistical analyses were performed using SAS software V9.4, SAS Institute Inc., Cary, NC, USA and R version 3.6.0, R foundation, Vienna, Austria.

## 3 RESULTS

In total, we identified 280 515 STEMI and 595 220 NSTEMI cases over the 4‐year period. STEMI cases decreased from 72 894 in 2014, to 70 230 in 2015, to 69 178 in 2016 and to 68 213 in 2017 with about 70% of STEMI cases assignable to male patients (supplemental Figure I). Key characteristics of patients are displayed in Table 1. Female sex was associated with markedly higher age (median [interquartile range {IQR}]: 74 (20) vs. 62 (19) years). In STEMI, there were 59.9% of females versus 33.1% of male cases aged ≥70 years; in NSTEMI 74.7% of female versus 54.0% of male cases were ≥ 70 years, respectively. Women and men differed also markedly regarding frequency of relevant co‐morbidities: Female cases had a higher incidence of chronic kidney disease, hypertension, left ventricular heart failure, or atrial fibrillation in STEMI as well as NSTEMI (Table 1).

**TABLE 1:** Baseline characteristics of patients hospitalized with AMI in 2014–2017

(A)STEMI		
	Male; *n* (% within subgroup)	Female; *n* (% within subgroup)
Total (% within subgroup)	196 177 (64.7)	84 338 (35.3)
Age, median (IQR) (yrs)	62 (19)	74 (20)
Anterior wall infarction, *n* (%)	93 903 (47.9)	40 693 (48.3)
Diabetes mellitus, *n* (%)	42 645 (21.7)	22 252 (26.4)
Chronic kidney disease, *n* (%)	24 548 (12.5)	16 157 (19.2)
Peripheral arterial disease 1–3, *n* (%)	4629 (2.4)	1939 (2.3)
Critical limb threatening ischemia, *n* (%)	1260 (0.6)	678 (0.8)
Atrial fibrillation, *n* (%)	25 552 (13.0)	14 864 (17.6)
Hypertension, *n* (%)	128 266 (65)	58 434 (69)
Stroke, *n* (%)	3827 (2.0)	2420 (2.9)
NYHA I, *n* (%)	6338 (3.2)	2091 (2.5)
NYHA II, *n* (%)	15 569 (7.9)	6149 (7.3)
NYHA III, *n* (%)	18 480 (9.4)	9272 (11.0)
NYHA IV, *n* (%)	22 852 (11.6)	12 830 (15.2)
Right ventricle chronic heart failure, *n* (%)	8964 (4.6)	6040 (7.2)
Chronic heart failure, *n* (%)	67 194 (34.3)	32 811 (38.9)
Cardiogenic shock, *n* (%)	23 102 (11.9)	11 272 (13.4)
Impella, *n* (%)	1202 (0.6)	398 (0.5)
ECMO, *n* (%)	1753 (0.9)	514 (0.6)
Dyslipidemia, *n* (%)	96 639 (49.3)	37 004 (43.9)
Obesity, *n* (%)	14 656 (7.5)	6766 (8.0)
Smoking, *n* (%)	23 674 (12.1)	6249 (7.4)
Cancer, *n* (%)	2648 (1.3)	1254 (1.5)

B) NSTEMI		
	Male; *n* (% within subgroup)	Female; *n* (% within subgroup)
Total (%)	384 905 (69.9)	210 315 (30.1)
Age, median (IQR) (yrs)	71 (19)	78 (15)
Anterior wall infarction, *n* (%)	982 (0.3)	479 (0.2)
Diabetes mellitus, *n* (%)	121 467 (31.6)	70 338 (33.4)
Chronic kidney disease, *n* (%)	92 120 (23.9)	62 485 (29.7)
Peripheral arterial disease 1–3, *n* (%)	18 892 (4.9)	7505 (3.6)
Critical limb threatening ischemia, *n* (%)	6150 (1.6)	2957 (1.4)
Atrial fibrillation, *n* (%)	85 155 (22.1)	55 400 (26.3)
Hypertension, *n* (%)	286 800(74.5)	162 870 (77.4)
Stroke, *n* (%)	10 993 (2.9)	6973 (3.3)
NYHA I, *n* (%)	8513 (2.2)	4024 (1.9)
NYHA II, *n* (%)	26 953 (7)	14 130 (6.7)
NYHA III, *n* (%)	45 608 (11.8)	27 279 (13)
NYHA IV, *n* (%)	48 583 (12.6)	31 975 (15.2)
Right ventricle chronic heart failure, *n* (%)	29 449 (7.7)	20 849 (9.9)
Chronic heart failure, *n* (%)	140 259 (36.4)	85 235 (40.5)
Cardiogenic shock, *n* (%)	14 803 (3.9)	7328 (3.5)
Impella, *n* (%)	745 (0.2)	299 (0.1)
ECMO, *n* (%)	982 (0.3)	479 (0.2)
Dyslipidemia, *n* (%)	183 490 (47.7)	85 150 (40.5)
Obesity, *n* (%)	32 214 (8.4)	17 352 (8.3)
Smoking, *n* (%)	27 438 (7.1)	7467 (3.6)
Cancer, *n* (%)	8758 (2.3)	3726 (1.8)

*Note*: Left ventricular failure (LVF) was stage‐related encoded to the New York Heart Association (NYHA) classification. Age given in median (interquartile range).

Abbreviations: IQR, interquartile range; NSTEMI, non–ST‐segment elevation myocardial infarction.

**FIGURE 1:**
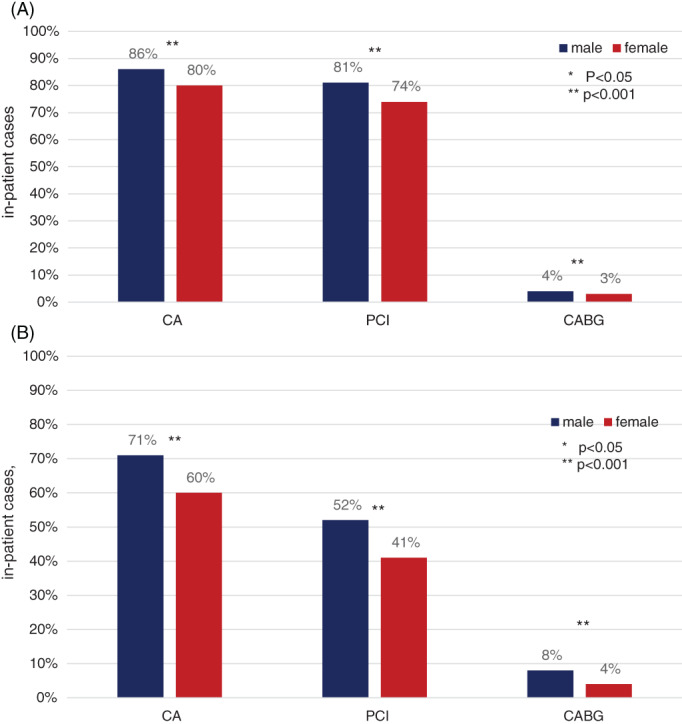
In‐hospital treatment dependent on sex in STEMI and NSTEMI. In‐hospital treatment of acute myocardial infarction 2014–2017: STEMI (panel A) and NSTEMI (panel B). With regard to therapeutic strategies, 74.3% of female and 81.3% of male STEMI cases received percutaneous coronary intervention (PCI; p < .001); coronary bypass surgery (CABG) was performed in 2.7% of female cases versus 4.2% of male (p < .001). In NSTEMI, PCI was performed in 40.8% of female and 52.0% of male cases (p < .001). CA, coronary angiography; CABG, coronary artery bypass surgery; NSTEM, non ST‐elevation myocardial infarction; PCI, percutaneous coronary intervention; STEMI, ST‐elevation myocardial infarction

**FIGURE 2:**
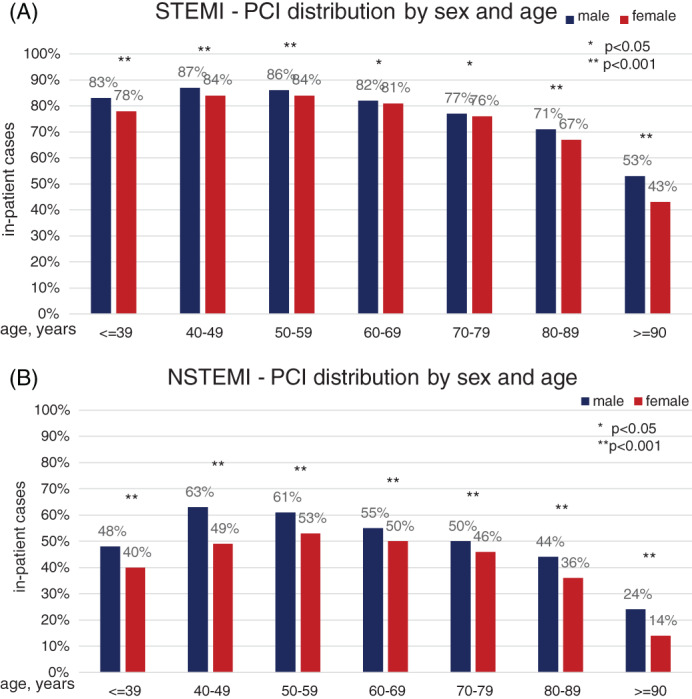
Age‐ and sex‐dependent application of PCI in STEMI and NSTEMI. The observed (bars) application of percutaneous coronary intervention (PCI) is given for female (red) and male (blue) cases for STEMI (panel A) and NSTEMI (panel B). The chance of receiving PCI decreases particularly in females at the upper bounds of the age distribution in both, STEMI and NSTEMI. Of the 5125 female and 2015 male STEMI patients aged 90 years and older, these received statistically notable less frequent percutaneous coronary intervention (42.5% female vs. 52.8% male; p < .001). NSTEMI, non‐ST‐elevation acute myocardial infarction; STEMI, ST‐elevation acute myocardial infarction

With regard to therapeutic strategies, 74.3% of female and 81.3% of male STEMI cases received percutaneous coronary intervention (PCI; p < 0.001). Coronary bypass surgery (CABG) was performed in 2.7% of female cases vs. 4.2% of male (p < .001; Figures 1 and 2). Of the 5125 female and 2015 male STEMI patients aged 90 years and older, these received statistically notable less frequent PCI (42.5% female vs. 52.8% male; p < .001) and CABG (0.1% female vs. 0.4% male; p < 0.001) compared to younger age groups. In NSTEMI, PCI was performed in 40.8% of female and 52.0% of male cases (p < .001).

### 3.1 In‐hospital mortality

**FIGURE 3:**
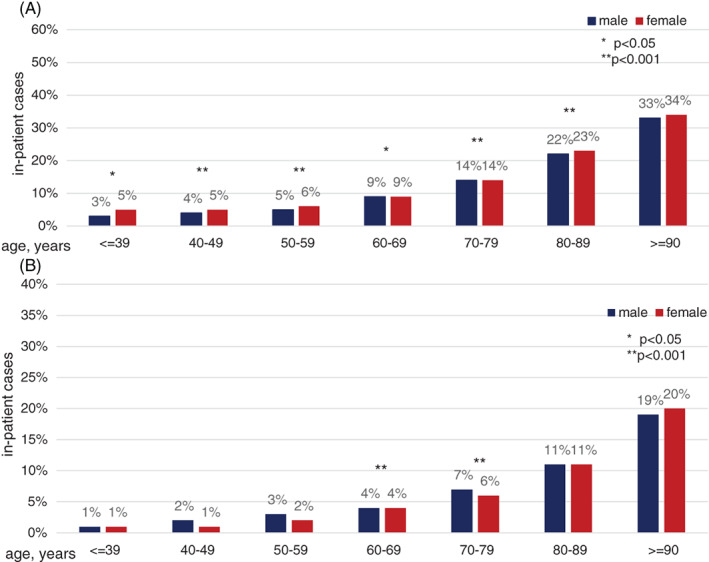
In‐hospital mortality dependent on sex. Observed (bars) data show in‐hospital mortality of STEMI (panel A) and NSTEMI (panel B). The observed in‐hospital mortality was notably increased in female compared to male patients with STEMI (15.0% vs. 9.6%; p < .001) and also in NSTEMI (8.4% vs. 6.3%; p < .001). NSTEM, non ST‐elevation myocardial infarction; STEMI, ST‐elevation myocardial infarction

**FIGURE 4:**
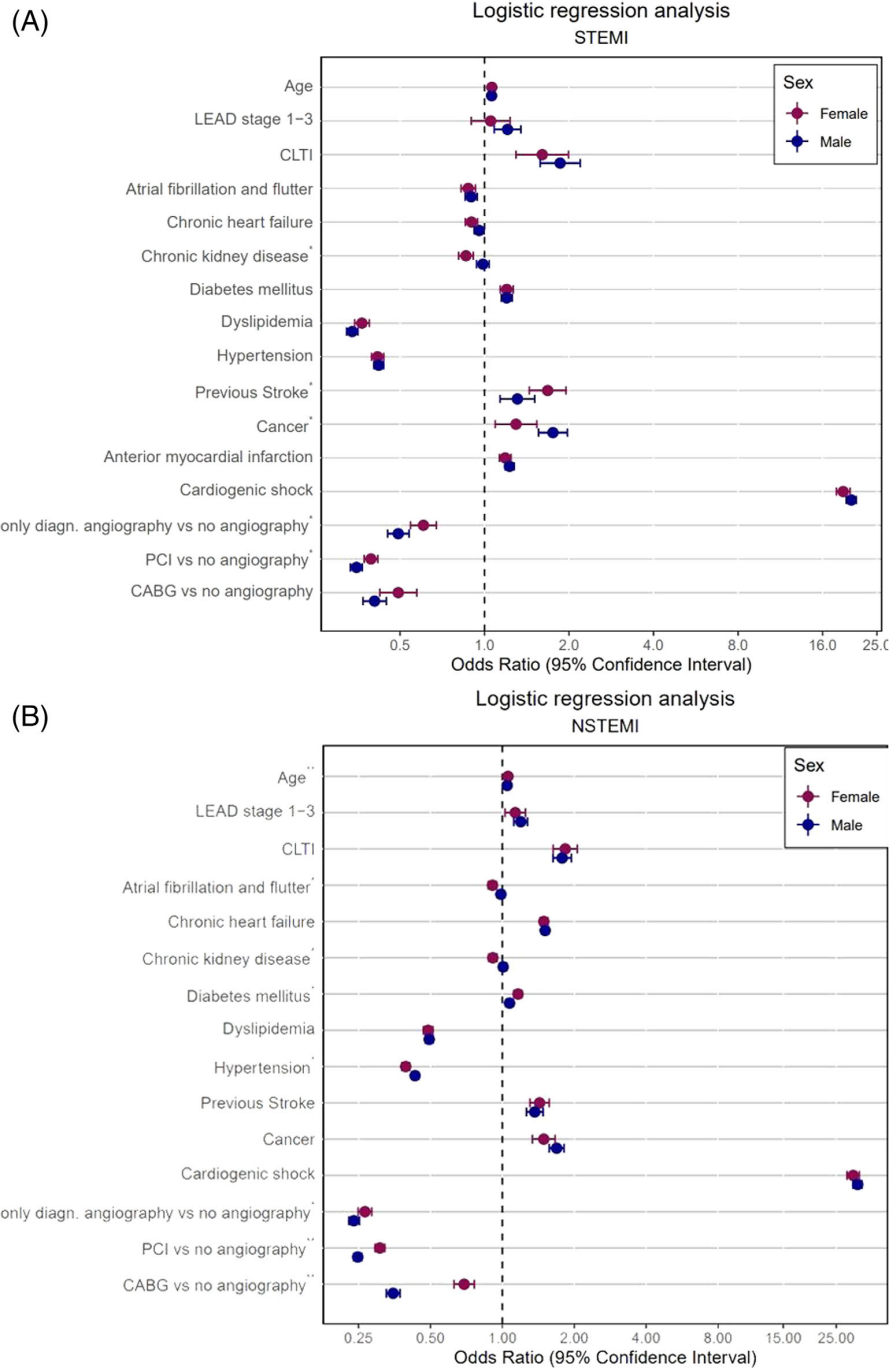
Forest‐plot of a multivariable logistic regression analysis for in‐hospital mortality of STEMI and NSTEMI. Multivariable logistic regression analysis for in‐hospital mortality of ST‐elevation myocardial (STEMI; panel A) and non ST‐elevation myocardial infarct (NSTEMI; panel B) was performed to evaluate the association of sex and in‐hospital death adjusted by patient's risk profile in a full model (including all patients) and for female and male sex separately. The models included age, diabetes mellitus (DM), chronic heart failure (CHF), chronic kidney disease (CKD), peripheral arterial disease (PAD), atrial fibrillation and/or flutter (Afib), hypertension, previous stroke, dyslipidemia, obesity, smoking, cancer and a disjoint categorical variable (no angiography, diagnostic angiography only without revascularization, PCI, CABG) to considered different treatment strategies

The observed in‐hospital mortality was notably increased in female compared to male patients with STEMI with 12 672 (15%) versus 18 817 (9.6%) cases (p < .001). This was similar in NSTEMI with 17 502 (8.3%) versus 24 336 (6.3%) cases (p < .001); Figure 3). After adjustment for numerous co‐factors, female sex remained an independent factor for increased in‐hospital mortality in STEMI (OR 1.07; 95% CI 1.03–1.10; p < .001), while it was associated with lower mortality in NSTEMI (OR 0.91; 95% CI 0.89–0.93; p < .001). The outcome of AMI was also significantly altered by the patients' co‐morbidity as indicated by the encoded secondary diagnoses (Figure 4 and supplemental Table I).

Multivariable logistic regression analyses adjusted by patient's risk profile for in‐hospital death showed that chronic heart failure was associated with an decreased in‐hospital mortality in STEMI and an increased in‐hospital mortality in NSTEMI (STEMI: male OR 0.96; 95% CI 0.92–1.00; female OR 0.90; 95% CI 0.86–0.95; NSTEMI: male OR 1.51; 95% CI 1.46–1.56; female OR 1.49; 95% CI 1.44–1.55). A significant difference between women and men could not be shown (STEMI p^int^ = .106; NSTEMI p^int^ = .713).

Age was associated with an increased in‐hospital mortality in STEMI and NSTEMI in both sexes, but a significant difference between men and women could only be seen in NSTEMI cases (p^int^ < .001).

The use of PCI in comparison to no angiography was associated with an decreased in‐hospital mortality in case of NSTEMI (female: OR 0.31; 95% CI 0.29–0.32; male: OR 0.25; 95% CI 0.24–0.26) as well as in case of STEMI (female: OR 0.39; 95% CI 0.37–0.42; male: OR 0.35; 95% CI 0.33–0.37). A significant difference between men and women could be shown in case of STEMI (p^int^ = .006) as well as NSTEMI (p^int^ < .001; Figure 4).

In addition, the use of CABG in comparison to no angiography decreased in‐hospital mortality in both sexes (STEMI: women OR 0.49; 95%‐CI 0.42–0.57 men OR 0.41; 95% CI 0.37–0.45; NSTEMI women OR 0.69, 95% CI 0.63–0.76; men OR 0.35; 95% CI 0.33–0.37). A significant difference between men and women could only be shown in case of NSTEMI (p^int^ < .001).

### 3.2 Costs and length of hospital stay

The average (± standard deviation [STD]) cost per STEMI case was 7240 (±10 725) EUR in male and 6511 (±9181) EUR in female patients, and 6376 (±9398) EUR per male and 5367 (±7505) EUR per female NSTEMI case. The mean (STD) length of hospital stay for AMI was 8 (±8) days in male STEMI and 8 (±8) days in female STEMI and 8 (±8) days in male and 8 (±8) days in female NSTEMI cases.

## 4 DISCUSSION

In this nationwide real‐world setting including all AMI cases from Germany of the years 2014 to 2017, in‐patient STEMI cases continue to decrease over the recent past in both, male and female patients. Furthermore, our data showed, that there were not only marked differences in baseline characteristics of in‐patient STEMI and NSTEMI cases but also regarding the in‐hospital treatment strategies between female and male patients. The manifestation of AMI in female patients occurred on average at an older age compared to men. Higher age and more frequent unfavorable comorbidities of women are discussed as the major cause for the higher in‐hospital mortality in comparison to men. Moreover, the American Heart Association recommends to improve the under‐representation of women in clinical trials due to the fact that data presented with sex‐ and gender‐specific results are currently lacking in many studies.^20^

### 4.1 In‐hospital mortality and impact of comorbidities

In our study, the observed in‐hospital mortality was notably increased in female patients with STEMI and NSTEMI compared to males. After adjustment to various influencing factors, female sex independently increased in‐hospital mortality by almost 7% in the case of STEMI but was associated with decreased mortality in the case of NSTEMI. Previous published data from the *Swedish Web System for Enhancement of Evidence‐Based Care in Heart Disease Evaluated According to Recommended Therapies* (SWEDEHEART) registry reported a similar trend, with a higher short‐term mortality in female STEMI patients. This trend was driven by a high risk of cardiogenic shock in the pre‐hospital‐phase, which is not covered in our analysis. In their analyses, the authors concluded that the trend was age‐dependent.^21^ A French study analyzed data from 74 389 patients hospitalized with acute myocardial infarction. Female patients had a higher rate of hospital mortality (14.8% versus 6.1%; p < .0001). As in our study, women in the French study were on average older (75 versus 63 years of age; p < .001).^22^ Nevertheless, there are also studies without any difference concerning in‐hospital mortality due to gender. In a Polish nationwide study cohort of patient with AMI, female sex did not increase the in‐hospital mortality (OR 0.97).^23^

### 4.2 Impact of in‐hospital treatment

Our results confirm prior studies,^24^, ^25^ which showed that women presenting with acute coronary syndrome were less likely to undergo diagnostic catheterization as well as PCI than men although various studies have demonstrated a benefit from PCI for women and an association with a decreased in‐hospital mortality.^26^, ^27^, ^28^

In our study, the use of PCI was associated with a decreased in‐hospital mortality in case of STEMI as well as NSTEMI cases in both sexes. In contrast to this, studies such as FRISC‐II and RITA‐3‐trial that concluded that women with NSTEMI do not benefit significantly from an early invasive treatment in comparison to a conservative treatment.^29^, ^30^ In addition, a large study with 11 931 consecutive patients who underwent PCI for various indications during 2000–2009, showed that women undergoing PCI for STEMI had higher mortality than men. This could only partially be explained by a difference in baseline characteristics with more frequent unfavorable co‐morbidities.^31^

Female sex was also associated with decreased n‐hospital mortality in case of CABG surgery. In the CADILLAC trial, the authors explain the higher mortality in women after interventional treatment by differences in lower body size as well as more unfavorable clinical risk factors.^32^ Reinecke et al. showed in their analyze of clinical and procedural data of 6681 patients who underwent PTCA and subsequent emergency CABG from 1989 to 1998 that women had an increased risk for failure of PTCA and a markedly higher operative mortality after emergency CABG. However, in multivariate analyses, female gender was not an independent predictor of postoperative death.^33^ In addition, some studies of coronary‐artery bypass surgery show increased perioperative mortality rate for women than men due to narrower coronary arteries, which may lead to more technical difficulties as well as less complete revascularization and lower success rates compared to men. However, long‐term survival turned out to be similar.^34^, ^35^, ^36^

One of many possible influencing factors explaining the differences may be a sex bias in the delivery of medical care. This could mean, that women undergo procedures later or men undergo procedures at a time when the disease is significantly less advanced and the outcome due to conservative care might not show a difference.

We also found an association between age and sex: differences due to the rate of invasive treatment (at least one out of diagnostic catheterization, PCI and/or CABG) widened with increasing age. A similar effect could be shown by Gan et al., who found that age was associated with an increased in‐hospital mortality in STEMI and NSTEMI in both sexes, but a significant difference between men and women could only be seen in NSTEMI cases.^15^

### 4.3 Impact of costs and length of hospital stay

Both costs and length of stay are influenced by the patient's morbidity, diagnoses and procedures. Furthermore, due to the principles of the German DRG system, the length of the hospital stay has also impact on the costs incurred. Since women received less often interventions, this could be one reason why the total cost of stay was lower than that of men with an average of the same length of stay.

### 4.4 Strengths and limitations

One of the strengths of the presented data is the large‐sized, unselected population of the entire nation which not only represent individual cases but allows us to analyze the real situation. The reliability and validity of the used ICD and OPS codes is high, because they directly impact the hospitals reimbursement. Furthermore, due to this high impact in reimbursement, all diagnostic and procedural codes are independently verified by the MDK (Medizinischer Dienst der Krankenversicherung/Medical Service of the Health Insurance) in more than 20%–30% of cases to assure that they are correct.

However, there are also some limitations of the study. The data were not patient‐ but case‐based, which could result in some patients being counted twice in a year; however, this could only lead to underestimation of in‐hospital mortality. In addition, the fact that the database is administrative and not clinical is also a limitation. Another limitation is the fact that the DESTATIS reflects only the hospital stay and any information after discharge is missing. In addition, these administrative data did not contain all clinically important information which may influence the outcome. The high percentage of patients who were coded as having NYHA IV heart failure may seem raise further questions about data reliability and could at least partly be due to the fact that people with heart failure NYHA IV may be transferred to other hospitals more often and are therefore counted twice in our analysis.

## 5 CONCLUSION

Our nationwide real‐world data documented that the observed increased in‐hospital mortality in female patients could largely attributed to the impact of the more unfavorable comorbidities and age, rather than due to female‐intrinsic factors. With regard to standard coronary treatments (either diagnostic coronary angiography or PCI), female sex were associated with a decreased in‐hospital mortality in STEMI and NSTEMI cases. Of note, female patients, and especially older women, received major diagnostic and therapeutic procedures less frequently than men.

Further studies will be needed to determine whether the differences in procedural rates reflect appropriate clinical practice or whether the outcome of women with acute coronary syndrome are negatively affected by these differences. Therefore, it is important that health care providers are made aware of potential sex differences in treatment, particularly in regard to the use of diagnostic catheterization in older patients.

## CONFLICT OF INTEREST

Lena Makowski reports other from Bayer Vital, outside the submitted work. Jannik Feld, Jeanette Koeppe, Julia Illner, Leonie Kuehnemund, Achim Wiederhold and Joachim Gerß have nothing to disclose. Holger Reinecke reports personal fees from Daiichi, grants from BMS/Pfizer, personal fees from MedUpdate, personal fees from DiaPlan, personal fees from NeoVasc, grants and personal fees from Pluristem, grants from Bard, grants from Biotronik, personal fees from NovoNordisk, outside the submitted work. Eva Freisinger reports non‐financial support from BAYER, non‐financial support from Vascuros, outside the submitted work.

## ETHICS STATEMENT

The data present here was assessed in the GenderVasc research project. This project was approved by the Ethics Committee of the Landesaerztekammer Westfalen‐Lippe and the Medical Faculty of the Westfaelische Wilhelms‐University Muenster (No 2019‐21‐f‐S).

## Supporting information


**Appendix S1**: supporting information

## Data Availability

The authors confirm that the data utilized in this study cannot be made available in the manuscript, the supplemental files, or in a public repository due to German data protection laws (‘Bundesdatenschutzgesetz’, BDSG). Generally, access to data of statutory health insurance funds for research purposes is possible only under the conditions defined in German Social Law (SGB V § 287). Requests for data access can be sent as a formal proposal specifying the recipient and purpose of the data transfer to the appropriate data protection agency. Access to the data used in this study can only be provided to external parties under the conditions of the cooperation contract of this research project and after written approval by the sickness fund.
