# Supplementary material for: Gender differences in acute myocardial infarction—A nationwide German real‐life analysis from 2014 to 2017
Source: Clin Cardiol. 2021 Jun 1;44(7):890–8. doi: 10.1002/clc.23662 (PMC8259152; doi:10.1002/clc.23662)

Appendices – Supplementary

Appendix Figure Legends

Appendix Figure 1: In-patient cases of acute myocardial infarction in 2014-2017

In-hospital cases of acute myocardial infarction 2014-2017: STEMI (panel A) and NSTEMI (panel B). In total, we identified 280,515 STEMI and 595,220 NSTEMI cases over the 4-year period. STEMI cases decreased from 72,894 in 2014, to 70,230 in 2015, to 69,178 in 2016 and to 68,213 in 2017.

Appendix Table 1: Diagnoses and Procedure Codes

Diagnosis	ICD 10
**Acute Myocardial Infarction**	**I21 – I22**
ST- segment elevation myocardial infarction	I21.0, I21.1, I21.2, I22.0, I22.1, I22.8
Non ST- segment elevation myocardial infarction	I21.3, I21.4, I21.9, I22.9
Secondary Diagnosis	
**Acute anterior Wall infarction**	I21.0, I22.0
**Chronic Kidney Disease**	N18, N19
**Diabetes Mellitus**	E10-E14
**Hypertensive Disease**	I10-I15
**Left Ventricular Failure**	I50.11-I50.14
NYHA I	I50.11
NYHA II	I50.12
NYHA III	I50.13
NYHA IV	I50.14
**Right Ventricular Failure**	I50.0
**Cardiogenic Shock**	R.57.0
**Chronic Heart Failure (LVHF or RVHF)**	I50
**Atrial Fibrillation and/ or flutter**	I48
**Acute Stroke**	I63, I64
**Previous stroke**	I69.3, I69.4
**Dyslipidemia**	E78*

**Obesity**	E66*
**Smoking**	F17*
**Cancer**	C*
**Lower extremity arterial disease (LEAD)**	
LEAD 1-3	I70.20, I70.21<br>Since 2015: I70.20, I70.21, I70.22
LEAD 4-5	I70.22, I70.23, I70.24<br>Since 2015: I70.23, I70.24, I70.25
Procedures	OPS code
**Coronary angiography**	1-275
**Percutaneous coronary intervention**	8-837
**Coronary artery bypass surgery**	5-36
**Impella**	8-83a.3
**ECMO**	8-852.3

AMI as principal diagnosis and co-morbidities as secondary diagnoses were coded according to the German Modification of the International Statistical Classification of Diseases and Related Health Problems 10th Revision (ICD-10-GM). Procedures were coded according to the German Procedure Classification (OPS).

Appendix Table 2: Results of the logistic regression analysis

STEMI						
Covariable	female			male		
	Odds ratio	95%-Confidence Interval	p-value	Odds ratio	95%-Confidence Interval	p-value
Age	1.06	1.06 – 1.07	<.001	1.06	1.06 – 1.06	<.001
LEAD stage 1-3	1.05	0.90 – 1.23	0.532	1.21	1.08 – 1.35	<.001
CLTI	1.61	1.30 – 1.99	<.001	1.86	1.58 – 2.19	<.001
Atrial fibrillation and flutter	0.88	0.83 – 0.93	<.001	0.90	0.85 – 0.94	<.001
Chronic heart failure	0.90	0.86 – 0.95	<.001	0.96	0.92 – 1.00	0.033
Chronic kidney disease	0.86	0.81 – 0.91	<.001	0.99	0.94 – 1.04	0.618
Diabetes mellitus	1.20	1.14 – 1.27	<.001	1.20	1.15 – 1.26	<.001
Dyslipidemia	0.37	0.35 – 0.39	<.001	0.34	0.32 – 0.35	<.001
Hypertension	0.42	0.40 – 0.44	<.001	0.42	0.40 – 0.44	<.001
Previous Stroke	1.68	1.44 – 1.95	<.001	1.31	1.14 – 1.26	<.001
Cancer	1.30	1.09 – 1.54	0.003	1.75	1.56 – 1.97	<.001
Anterior myocardial infarction	1.19	1.13 – 1.24	<.001	1.23	1.18 – 1.28	<.001
Cardiogenic shock	18.95	17.92 – 20.04	<.001	20.26	19.44 – 21.11	<.001
Only diagn. angiography vs no angiography	0.61	0.55 – 0.67	<.001	0.49	0.45 – 0.54	<.001
PCI vs no angiography	0.39	0.37 – 0.42	<.001	0.35	0.33 – 0.37	<.001
CABG vs no angiography	0.49	0.42 – 0.57	<.001	0.41	0.37 – 0.45	<.001

NSTEMI						
Age	1.06	1.05 – 1.06	<.001	1.05	1.05 – 1.05	<.001
LEAD stage 1-3	1.13	1.03 – 1.25	0.013	1.19	1.12 – 1.28	<.001
CLTI	1.83	1.63 – 2.06	<.001	1.78	1.63 – 1.94	<.001
Atrial fibrillation and flutter	0.91	0.87 – 0.95	<.001	0.99	0.95 – 1.02	0.422
Chronic heart failure	1.49	1.44 – 1.55	<.001	1.51	1.46 – 1.56	<.001
Chronic kidney disease	0.91	0.88 – 0.95	<.001	1.01	0.97 – 1.04	0.651
Diabetes mellitus	1.16	1.12 – 1.21	<.001	1.07	1.04 – 1.11	<.001
Dyslipidemia	0.49	0.47 – 0.51	<.001	0.49	0.48 – 0.51	<.001
Hypertension	0.39	0.38 – 0.41	<.001	0.43	0.42 – 0.45	<.001
Previous Stroke	1.43	1.31 – 1.57	<.001	1.37	1.26 – 1.48	<.001
Cancer	1.49	1.34 – 1.66	<.001	1.69	1.57 – 1.81	<.001
Cardiogenic shock	29.44	27.74 – 31.25	<.001	30.71	29.43 – 32.05	<.001
Only diagn. angiography vs no angiography	0.27	0.25 – 0.28	<.001	0.24	0.23 – 0.25	<.001
PCI vs no angiography	0.31	0.29 – 0.32	<.001	0.25	0.24 – 0.26	<.001
CABG vs no angiography	0.69	0.63 – 0.76	<.001	0.35	0.33 – 0.37	<.001

Appendix Table 3: Interaction p-value

Estimates, unadjusted and FDR-corrected p-values for the interaction estimates of the interaction of the features with sex for the multivariable logistic regression models. Effects of interaction were tested using type 3 effect-test.

Covariable	STEMI			NSTEMI		
	Estimate of interaction with sex (β)	Unadjusted interaction p-value	FDR-corrected interaction p-value	Estimate of interaction with sex (β)	Unadjusted interaction p-value	FDR-corrected interaction p-value
Age	0.002	0.236	0.361	0.008	<.001	<.001
LEAD stage 1-3	-0.138	0.160	0.262	-0.052	0.391	0.505
CLTI	-0.147	0.288	0.388	0.030	0.690	0.764
Atrial fibrillation and flutter	-0.023	0.554	0.660	-0.082	0.002	0.007
Chronic heart failure	-0.063	0.055	0.106	-0.012	0.621	0.713
Chronic kidney disease	-0.139	<.001	0.003	-0.010	<.001	0.002
Diabetes mellitus	-0.001	0.988	0.988	0.082	0.002	0.006
Dyslipidemia	0.082	0.032	0.068	-0.009	0.753	0.805
Hypertension	-0.009	0.784	0.810	-0.091	<.001	0.002
Previous Stroke	0.248	0.019	0.045	0.046	0.461	0.572
Cancer	-0.303	0.004	0.012	-0.124	0.063	0.108
Anterior myocardial infarction	-0.036	0.245	0.361	**	**	**
Cardiogenic shock	-0.067	0.060	0.108	-0.042	0.258	0.363
Only diagn. angiography vs no angiography	0.205	0.004	0.011	0.106	0.013	0.034
PCI vs no angiography	0.120	0.002	0.006	0.211	<.001	<.001

CABG vs no angiography	0.195	0.033	0.068	0.684	<.001	<.001

Appendix Table 4:

Differences between female and male sex were tested via two-sided Fischer's exact test or two-sided Chi-Square test, in the case of too large dataset.

1. **Death: total n (%)**						
Age (years)	STEMI			NSTEMI		
	female	male	p-value	female	male	p-value
All age	12,672 (15.0%)	18,817 (9.6%)	<.001	17,502 (8.3%)	24,336 (6.3%)	<.001
<=39	54 (5.1%)	158 (3.5%)	0.015	15 (1.3%)	64 (1.5%)	0,680
40-49	226 (4.8%)	832 (3.6%)	<.001	83 (1.5%)	386 (1.7%)	0,244
50-59	702 (5.8%)	2,766 (5.1%)	<.001	407 (2.47%)	1,645 (2.6%)	0,579
60-69	1,445 (9.0%)	4,169 (8.5%)	0.046	1,178 (3.9%)	3,669 (4.3%)	0,008
70-79	3,462 (14.5%)	5,815 (13.5%)	<.001	4,040 (6.2%)	8,108 (6.8%)	<.001
80-89	5,044 (23.5%)	4,412 (22.3%)	0.005	8,004 (11.0%)	8,578 (11.0%)	0,961
>=90	1,739 (33.9%)	665 (33.0%)	0,.470	3,775 (20.2%)	1,886 (19.4%)	0,094

2. PCI: total n (%)						
Age (years)	STEMI			NSTEMI		
	female	male	p-value	female	male	p-value
All age	62,692 (74.3%)	159,522 (81.3%)	<.001	85,732 (40.8%)	200,021 (52.0%)	<.001
<=39	826 (77.6%)	3,821 (83.4%)	<.001	474 (39.7%)	2,078 (48.4%)	<.001
40-49	3,950 (83.7%)	20,041 (86.8%)	<.001	2,765 (49.4%)	14,203 (63.1%)	<.001
50-59	10,059 (83.7%)	46,839 (85.8%)	<.001	8,754 (53.0%)	39,439 (61.1%)	<.001
60-69	13,055 (81.4%)	40,382 (82.3%)	0,010	14,967 (49.9%)	47,418 (55.4%)	<.001
70-79	18,270 (76.4%)	33,285 (77.3%)	0,014	29,896 (45.5%)	60,221 (50.1%)	<.001
80-89	14,353 (66.9%)	14,091 (71.3%)	<.001	26,348 (36.3%)	34,352 (44.1%)	<.001
>=90	2,179 (42.5%)	1,063 (52.8%)	<.001	2,528 (13.5%)	2,310 (23.7%)	<.001

3. Diagnostic coronary angiography: total n (%)						
Age (years)	STEMI			NSTEMI		
	female	male	p-value	female	male	p-value
All age	67,039 (79.5%)	168,043 (85.7%)	<.001	126,601 (60.2%)	272,064 (70.7%)	<.001
<=39	923 (86.8%)	4,046 (88.3%)	0,174	827 (69.2%)	2,992 (69.7%)	0,749
40-49	4,206 (89.2%)	20,695 (89.7%)	0.284	4,157 (74.3%)	17,584 (78.1%)	<.001
50-59	10,634 (88.5%)	48,531 (88.9%)	0,195	12,585 (76.2%)	50,149 (77.6%)	<.001
60-69	13,838 (86.3%)	42,498 (86.6%)	0,293	22,174 (73.9%)	64,175 (74.9%)	<.001
70-79	19,644 (82.2%)	35,856 (83.2%)	<.001	45,174 (68.8%)	85,817 (71.4%)	<.001
80-89	15,445 (71.9%)	15,255 (77.2%)	<.001	38,135 (52.5%)	48,279 (61.96%)	<.001
>=90	2,349 (45.8%)	1,162 (57.7%)	<.001	3,549 (18.9%)	3068 (31.48%)	<.001

4. CABG: total n (%)						
Age (years)	Age (years)			Age (years)		
	female	male	p-value	female	male	p-value
All age	2239 (2.65%)	8261 (4.21%)	<.0001	8548 (4.06%)	29493 (7.66%)	<.0001
<=39	22 (2.07%)	68 (1.48%)	0,1744	36 (3.01%)	116 (2.70%)	0,5506
40-49	73 (1.55%)	531 (2.3%)	0.001	181 (3.24%)	1266 (5.62%)	<.0001
50-59	298 (2.48%)	1924 (3.52%)	<.0001	741 (4.49%)	5143 (7.96%)	<.0001
60-69	582 (3.63%)	2494 (5.08%)	<.0001	1926 (6.42%)	8626 (10.07%)	<.0001
70-79	940 (3.93%)	2571 (5.97%)	<.0001	4053 (6.17%)	11087 (9.23%)	<.0001
80-89	320 (1.49%)	665 (3.37%)	<.0001	1596 (2.20%)	3215 (4.13%)	<.0001
>=90	4 (0.08%)	8 (0.4%)	0,0063	15 (0.08%)	40 (0.41%)	<.0001

5. Stroke: total n (%)						
Age (years)	STEMI			NSTEMI		
	female	male	p-value	female	male	p-value
All age	1,219 (1.45%)	1,996 (1.0%)	<.0001	2,469 (1.2%)	3.731 (1.0%)	0,392
<=39	7 (0.7%)	12 (0.3%)	0.070	5 (0.4%)	12 (0.3%)	0,274
40-49	36 (0.8%)	110 (0.5%)	0.020	26 (0.5%)	81 (0.4%)	0,909
50-59	112 (0.9%)	380 (0.7%)	0,008	98 (0.6%)	379 (0.6%)	0,105
60-69	202 (1.3%)	520 (1.1%)	0,037	311 (1.0%)	796 (0.9%)	0,008
70-79	412 (1.7%)	668 (1.6%)	0,090	921 (1.4%)	1508 (1.3%)	0,007
80-89	384 (1.8%)	287 (1.5%)	0,007	931 (1.3%)	879 (1.1%)	0,163
>=90	66 (1.3%)	19 (0.9%)	0,275	177 (1.0%)	76 (0.8%)	0,392

Appendix Figure 1:

A)

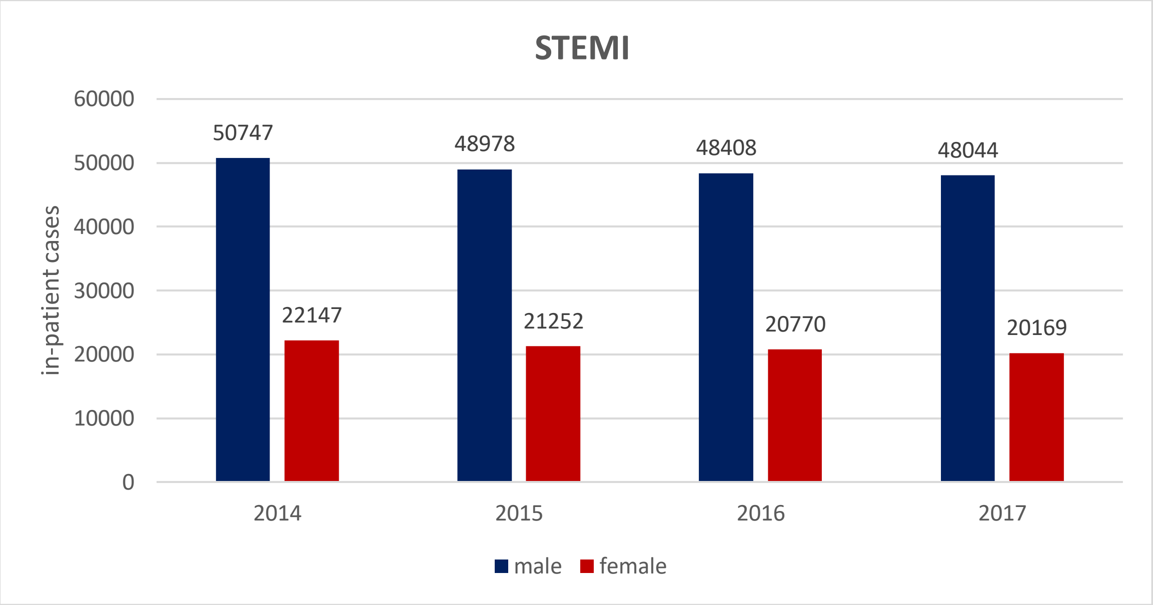

B)

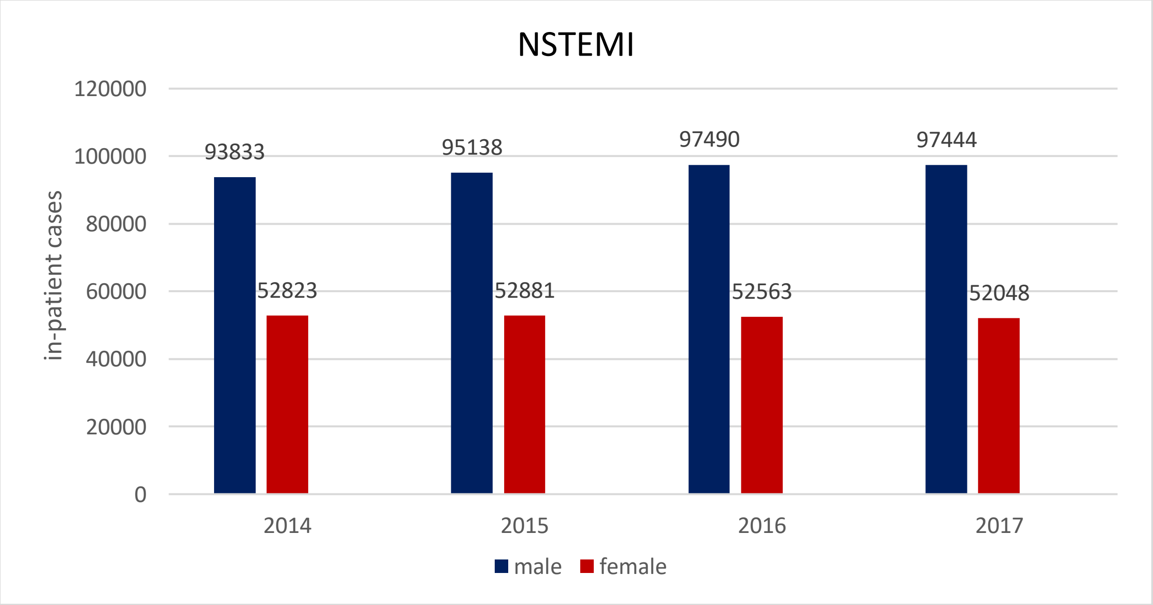

Supplement: Supplementary file 1 — Appendix S1: supporting information [file CLC-44-890-s001.pdf]
